# 

**Published:** 1873-10

**Authors:** 


### Contents of October Number.

### ORIGINAL DEPARTMENT.

ART. I.—Amputation at the Shoulder Joint. Remarks and Cases. By J. F. Miner, M. D. .... 81
ART. II.—A Case Showing the Danger of Error in Diagnosis. By Milton G. Potter, M. D., Prof. of Anatomy, etc. .... 83
ART. III.—Puncturing the Bladder above the Pubis. By W. C. Raymond, M. D. .... 88
ART. IV.—A Case of Colles Fracture with Luxation of the Ulna. By Charles D. Buckley, M. D. .... 89

### MISCELLANEOUS.

Foul Wells as a Cause of Typhoid Fever .... 90
On the use of Chlorate of Potash and Glycerine Injections for Ulcerations in Chronic Dysentery .... 91
A Case of Loss of Nearly Half of the Bones of the Skull, with Exposure and Sloughing of a Portion of the Brain .... 95
Rendering Hard Waters Soft .... 100
Rules for the Management of Infants .... 101
Nervous Diseases of Women .... 104
The Application of Earth in Surgery .... 106
Case of Recovery after Ruptured Uterus with Protrusion of Intestine .... 108

### EDITORIAL DEPARTMENT.

The Water Supply of Buffalo .... 109
Items and Remarks .... 113

BOOKS REVIEWED:

Insanity and its Relations to Crime. A Text and a Commentary. By Wm. A. Hammond, M. D. .... 116

Books and Pamphlets Received .... 120

